# Is central line type an independent risk factor of central line-associated bloodstream infection in a neonatal intensive care unit population? Experiences at a pediatric hospital in South Texas

**DOI:** 10.1017/ash.2023.534

**Published:** 2024-01-31

**Authors:** Danielle J. Durant, Nancy Fallwell, Lesley Martinez, Claudia Guerrazzi-Young

**Affiliations:** 1 College of Nursing and Health Sciences, Texas A&M University—Corpus Christi, Corpus Christi, TX, USA; 2 Behavioral Health & Health Policy Practice, Westat, Inc., Rockville, MD, USA; 3 Infection Prevention and Control Department, Driscoll Children’s Hospital, Corpus Christi, TX, USA; 4 Jack C. Massey College of Business, Belmont University, Nashville, TN, USA

## Abstract

**Background:**

Central line-associated bloodstream infection (CLABSI) causes significant harm in neonatal intensive care unit (NICU) patients. However, data regarding risk factors and prevention strategies for CLABSI in NICU patients is limited.

**Objective:**

To examine risk factors for CLABSI in a NICU population, with particular interest in central line type and site placement.

**Design:**

Retrospective case–control study.

**Setting:**

NICU (Level IV, 67 bed) at a pediatric hospital in South Texas.

**Participants:**

All central line insertions and subsequent CLABSI cases were extracted from the EHR for NICU admissions occurring from January 1, 2018, to November 3, 2022 (*N* = 1,356), along with potential CLABSI risk factors.

**Methods:**

Central line insertions resulting in CLABSI (*N* = 35) were compared to instances without CLABSI (*N* = 1,321) using bivariate and multivariate analysis, with propensity score matching.

**Results:**

Multivariate risk factors include implantable device (odds ratio [OR] = 14.5, *P* < .001), neck site placement (OR = 7.2, *P* < .001), and device dwell time (OR = 5.6, *P* = .001), as well as years 2021 (OR = 5.1, *P* = .017) and 2022 (OR = 5.9, *P* = .011). This indicates the odds of contracting CLABSI are 14.5 times higher when an implantable central line is used compared to the reference category (PICC devices). When cases are paired with matched controls, likelihood of CLABSI is 7.1% higher in patients with an implantable device than in similar patients with other central lines (*p* = 0.034).

**Conclusions:**

Implantable central lines are an independent risk factor for CLABSI in NICU patients at this facility.

## Introduction

Central line-associated bloodstream infections (CLABSI) are an important cause of morbidity and mortality in neonates.^1^ Premature infants have compromised immune systems and are often subject to invasive procedures, which increase their risk of CLABSI due to frequent central line use for delivering medications and parenteral nutrition, requiring daily device manipulations.^2^ However, according to the Centers for Disease Control and Prevention (CDC), although much evidence exists related to risk factors and prevention strategies for CLABSI in older children and the adult population, data regarding CLABSI in neonatal intensive care unit (NICU) patients is more limited and nuanced.^3^

Some risk factors for CLABSI in a NICU population have been established in previous studies, with birth weight^4–7^ and device dwell time^7,8^ the most cited. Additionally, use of parenteral nutrition^1,6,7,9^ and if the patient underwent surgery^1,2,10^ are associated with the incident of CLABSI in the NICU, as well as gestational age.^5^ Device type has also been found to be a risk factor, though the findings are inconsistent, with some studies implicating peripherally inserted central catheters (PICC),^4,8^ while others identify central venous catheters (CVC)^4^ and umbilical venous catheters (UVC)^7^ as significant. Other, less-cited, risk factors include the length of hospital stay; number of device lumens; receipt of blood products; device placement site—specifically, internal jugular^1^; male sex; and number of heel punctures.^2^

Understanding the potential for these infections to lead to severe complications and death for neonates, and the limited and inconsistent nature of the evidence, particularly regarding device type and CLABSI risk, more research is needed if effective prevention strategies are to be developed. The objective of this work was to add to this literature, assessing risk factors for CLABSI in the NICU population at a pediatric hospital in South Texas, with particular interest in the relationship of central line type and site placement with CLABSI. We conducted a retrospective case–control study identifying NICU patients with a central line who contracted CLABSI and compared those to the population that did not contract CLABSI, for the purposes of identifying significant risk factors.

## Methods

### Data

#### Extraction

All records for central lines and subsequent CLABSI events were extracted for the 67-bed, level IV NICU (average daily census 42) for admissions occurring from January 1, 2018, to November 3, 2022. The hospital transitioned to an electronic health record (EHR) integrated infection prevention surveillance system in 2018. To ensure data integrity, we extracted records from this system only, rather than include data from the prior surveillance software.

A structured query language (SQL) report was developed and utilized as the mechanism for data retrieval from the EHR and EHR surveillance system. This query included retrievable fields stored in the EHR database, in addition to some calculated fields computed based on available data points. A total of 1,380 records were identified with record granularity determined by one record per central line.

Records were excluded from the dataset if the central line placement date could not be ascertained by either electronic data pull or manual record review, likely due to the device being placed at another facility (*N* = 24). A proportion of records were identified with null values for the variables of central line site, number of lumens, and central line type; we manually reviewed narratives of operative notes to populate these missing fields.

#### Dependent variable

Central line insertions resulting in CLABSI were identified as a case (*N* = 35), with all other placements noted as no CLABSI (*N* = 1,321). In accordance with the National Healthcare Safety Network (NHSN) definition, CLABSI was defined as a bloodstream infection not related to another site of infection and identified via a positive blood culture for a patient with a central line in place longer than 2 days.^11^

#### Independent variables

Central line type and site placement were extracted as variables of particular interest in this study. Central line types used at this facility include PICC (*N* = 833), UVC (316), CVC (112), and implantable devices (95). These were coded as four indicator variables and defined as follows.

- PICC
- UVC
- Nontunneled CVC
- Implantable devices—tunneled venous catheters and infusaports

Site placements at this facility include basilic (*N* = 323), brachial (144), cephalic (74), antecubital (26), femoral (249), popliteal (30), saphenous (26), internal jugular (89), external jugular (48), subclavian (10), common facial (2), facial (1), and umbilical (316). Given the large number of sites, and some used with rarity, these were further categorized into four site groups: arm (basilic, brachial, cephalic, antecubital), leg (femoral, popliteal, saphenous), neck (internal and external jugular), and umbilical (umbilical), and coded as four indicator variables.

#### Control variables

Extracted with each line placement instances were known and potential predictors of CLABSI. These included patient sex; gestational age; birthdate, to calculate age at admission; birthweight, further grouped into NHSN birthweight categories: (A) ≤750 g, (B) 751–1000 g, (C) 1001–1500 g, (D) 1501–2500 g, (E) >2500g; admission and discharge date and time, for calculating total length of stay (LOS) and creation of a categorical time variable (year) for assessing changes in CLABSI over time; whether the patient had surgery, received parenteral nutrition, or blood products; date and time when the central line was placed and removed, to calculate age at line placement, total line dwell days, and days hospitalized before line placement; and number of device lumens.

#### Statistical software

All statistical analyses were performed using Stata/SE-17, produced by StataCorp LLC.

### Bivariate analysis

We first compared the two populations, CLABSI cases (*N* = 35) and no CLABSI cases (*N* = 1,321), to determine significant risk factors at the bivariate level. Two-tailed difference in means and proportion testing was performed across the risk factors using the student’s t-test for numeric variables and the χ2 test for categorical variables, respectively. These factors were examined to better understand the study population and determine significant relationships to further explore in the multivariate analysis.

### Multivariate analysis

#### Independent variables

We further explored risk factors for CLABSI using multivariate logistic regression analysis using two models: **model 1** included variables for central line type and **model 2** included variables for site placement group. Central line type and site placement groups were highly correlated with one another, which required that they be modeled separately. The models included the independent variables central line type (model 1), omitting PICC as the reference category due to no significant relationship with CLABSI at the bivariate level and its correlation with UVC use; and site group (model 2), omitting the arm site group as the reference category due to no association with CLABSI at the bivariate level and its correlation with the leg site group. Given the difficulty of interpreting coefficients from logistic regression models, odds ratios (OR) were calculated; the OR is “the odds an outcome will occur given a particular exposure, compared to the odds of the outcome occurring in the absence of that exposure.”^12^

#### Control variables

These models also controlled for specific predictors of CLABSI. Age at line placement and total line dwell days were both strongly correlated with total LOS, so age at admission, LOS before line placement, and total line dwell days were utilized in the model instead of LOS. Gestational age was highly, negatively correlated with very low birth weight (an indicator variable for NHSN birthweight categories A, B, and C); as such, very low birth weight was used in the model in accordance with existing literature. Other variables included male sex, surgery patient, receipt of blood products, receipt of parenteral nutrition, and year variables, to assess how CLABSI changed over time.

### Propensity score matching

To reduce the effect of confounding in our observational study, we used a propensity score matching method.^13,14^ To estimate the propensity score, we used common risk factors for CLABSI in a NICU population established in previous studies: birth weight, gender, device dwell time, use of parenteral nutrition, and if the patient underwent surgery, as well as controlled for time changes. The “treatment” explored in the analysis reflected the results of the multivariate analysis.

The nearest-neighbor matching approach was employed to achieve a 1-to-1 match utilizing the predictive values within a designated caliper distance. This method selects an “untreated” subject whose propensity score is closest to that of the “treated” subject and ensures that the absolute difference in propensity scores of matched subjects falls below a predetermined threshold, known as the caliper distance.^15^ We chose a caliper distance of 0.025, which we believed was small enough to provide a closer match between “treated” and “untreated” subjects, reducing bias and improving the precision of estimates. To assess the sampling variability in the propensity score model and to account for heteroskedasticity, we used robust standard errors.

### Compliance with protection of human subjects

The research protocol was determined to meet requirements for expedited review and was approved by the hospital’s institutional review board.

## Results

### Bivariate analysis

As shown in Table 1, only 2.6% (*N* = 35) of total central line insertions (*N* = 1,356) were associated with a CLABSI at this facility. Age at line placement was significantly higher in CLABSI cases (*P* = 0.001), with CLABSI patients about 26.7 days older on average when the central line was placed. CLABSI cases were characterized by lower birthweights, with a significantly higher proportion (54.3%) of very low birthweight (weight categories A–C) observations in the CLABSI group compared to the no CLABSI group (34.2%) (*P* = .014). A greater proportion of CLABSI cases received blood products (94.3%), compared to the no CLABSI group (75%) (*P* = .009).

**Table 1.** Characteristics of full population of patients receiving a central line (January 1, 2018–November 3, 2022)

Patient characteristics	CLABSI (*N* = 35)	No CLABSI (*N* = 1,321)	*P* value
Male sex	68.6%	58.0%	0.210
Gestational age (weeks)^a^	31.1	32.9	0.051
Age at admission (days)	8.7	6.6	0.414
Age at line placement (days)	49.6	22.9	0.001
Weight categories^b^			
Weight category A	25.7%	13.3%	0.035
Weight category B	11.4%	8.0%	0.468
Weight category C	17.1%	12.9%	0.456
Weight category D	5.7%	23.6%	0.013
Weight category E	40.0%	42.2%	0.799
Very-low birth weight	54.3%	34.2%	0.014
**Care characteristics**			
Surgical patient	65.7%	61.6%	0.623
Received parenteral nutrition	97.1%	87.4%	0.083
Received blood products	94.3%	75.0%	0.009
Number of lumens			
Single	42.9%	32.7%	0.205
Double	57.1%	66.3%	0.262
Triple	0.0%	1.1%	0.532
Central line type			
PICC	54.29%	61.62%	0.379
UVC	11.43%	23.62%	0.092
CVC	8.57%	8.25%	0.946
Implantable	25.71%	6.51%	<.001
Site groups^c^			
Neck	32.4%	10.7%	<.001
Leg	23.5%	22.8%	0.916
Arm	32.4%	42.7%	0.229
Umbilical	11.8%	23.9%	0.100
Total LOS (days)	120.6	71.6	0.001
LOS before line placement (days)	41.6	16.8	0.001
Line dwell time (days)	43.7	21.2	0.002
**Time period**			
2018	14.29%	22.71%	0.239
2019	8.57%	20.89%	0.075
2020	14.29%	16.58%	0.718
2021	37.14%	19.91%	0.012
2022	25.71%	19.91%	0.397

a Gestational age in completed weeks gestation as recorded in the patient’s medical record.

b Variables defined by the NHSN birthweight categories as follows: (A) ≤750 g, (B) 751–1000 g, (C) 1001–1500 g, (D) 1501–2500 g, (E) >2500 g (very-low birth weight) = categories A–C.

c Body site of central line as indicated in patient’s medical record grouped as follows: Arm = basilic (*N* = 323), brachial (144), cephalic (74), antecubital (26); leg = femoral (249), popliteal (30), saphenous (26); neck = internal jugular (89), external jugular (48), subclavian (10), common facial (2), facial (1); umbilical = umbilical (316).

Additionally, implantable central line devices were used in a significantly greater proportion of the CLABSI observations (25.7%) than in the observations that did not contract CLABSI (6.51%) (*P* < .001); similarly, a significantly greater proportion of neck site group placements (32.4%) were associated with CLABSI than in the no CLABSI group (10.7%) (*P* < .001). Further, total LOS (*P* = .001), LOS before line placement (*P* = .001), and total line dwell days (*P* = .002) were all significantly longer in the CLABSI population than in the no CLABSI group, with CLABSI cases associated with a LOS 49 days longer, a hospital stay before line placement 24.8 days longer, and total line dwell time 22.5 days longer.

### Multivariate analysis

Table 2 reports the results of the two logistic regression models—with central line type (model 1) and site placement group (model 2). Reviewing the control variables across models, total line dwell days is positively associated with instance of CLABSI and significant (model 1: *P* = .001, model 2: *P* < .001), with an OR of 5.6 and 6.6 in models 1 and 2, respectively. This indicates a 1% increase in total line dwell days is associated with 5.6–6.6 times increased odds of contracting CLABSI.

**Table 2.** Parameter estimates for logistic regression models of predictors of central line-associated blood stream infection (CLABSI) in a NICU population

Variable	Model 1			Model 2		
	OR	(SE)	*P*	OR	(SE)	*P*
Age at admission	0.989	(0.013)	0.408	0.991	(0.013)	0.501
Male sex^a^	1.793	(0.727)	0.150	1.701	(0.683)	0.186
Very-low birth weight^b^	1.793	(0.719)	0.145	1.874	(0.780)	0.131
Surgery patient^c^	0.702	(0.339)	0.464	0.729	(0.348)	0.508
Receipt of parenteral nutrition^d^	1.702	(2.040)	0.657	1.773	(2.117)	0.631
Receipt of blood products^e^	2.068	(1.821)	0.409	1.934	(1.697)	0.452
Line dwell time^f^	5.637	(2.914)	0.001	6.632	(3.386)	<.001
LOS before line placement	1.004	(0.003)	0.156	1.004	(0.003)	0.218
Year 2019^g^	1.262	(1.037)	0.777	0.953	(0.770)	0.953
Year 2020^g^	2.471	(1.865)	0.231	2.130	(1.573)	0.306
Year 2021^g^	5.060	(3.438)	0.017	4.264	(2.814)	0.028
Year 2022^g^	5.912	(4.116)	0.011	4.910	(3.351)	0.020
Multilumen device^h^	1.563	(0.834)	0.403	1.120	(0.523)	0.808
UVC device^i^	2.291	(1.567)	0.225	–	–	–
CVC device^i^	2.784	(1.926)	0.139	–	–	–
Implantable device^i^	14.460	(9.541)	<.001	–	–	–
Leg site group^j^	–	–	–	1.045	(0.556)	0.934
Neck site placement group^j^	–	–	–	7.165	(3.850)	<.001
Umbilical site placement group^j^	–	–	–	2.746	(1.958)	0.157

a Indicator variable for male sex coded as 1 if male, 0 if otherwise.

b Indicator variable for NHSN weight categories A–C coded as 1 if birthweight is in categories A–C, 0 if otherwise.

c Indicator variable for if the patient had surgery coded as 1 if surgical patient, 0 if otherwise.

d Indicator variable for if the patient received parenteral nutrition coded as 1 if patient received parenteral nutrition, 0 if otherwise.

e Indicator variable for if the patient received blood products coded as 1 if received blood products, 0 if otherwise.

f Continuous variable that failed assumption of linearity; logarithmic transformation was performed.

g Indicator variables for time period; year 2018 was omitted as reference category.

h Indicator variable for devices with more than one lumen coded as 1 if the device was multilumen, 0 if otherwise.

i Indicator variables for type of central line; PICC device omitted as reference category.

j Indicator variables for central line placement site groups; arm site group placement omitted as reference category.

Regarding the independent variables, in model 1, having an implantable device is significantly, positively associated with CLABSI (*P* < .001), with an OR of 14.5; this indicates the odds of contracting CLABSI are 14.5 times higher when an implantable central line is used than when PICC devices are employed (reference category); for comparison, odds for UVC and CVC devices compared to PICCs are 2.3 and 2.8, respectively, and were not significant.

Additionally, in model 2, the neck site placement group is significantly, positively associated with CLABSI (*P* < .001), with an OR of 7.2; this indicates that the odds of contracting CLABSI are 7.2 times higher when the central line is placed in one of the areas included in the neck site placement group than in an area in the arm site placement group (reference category). For comparison, odds for leg and umbilical site placement groups compared to the arm site group are 1.0 and 2.7, respectively, and were not significant. This is likely driven by the fact that of the 95 implantable devices used in this sample, 93 were placed in one of the areas included in the neck site group, and implantable devices constituted 62% of all central line types placed in this group.

### Propensity score matching

Given the results of the logistic regression analysis, implantable device was used as the “treatment” of interest for the propensity score matching analysis. Our primary metric for this analysis was the average effect of the “treatment” (implantable central line) on the “treated” (patients who received the treatment), which is the difference between the likelihood of CLABSI for patients with implantable devices and those of similar patients with other catheter types. The results indicate the likelihood of CLABSI is 7.1% higher in patients with an implantable device than in similar patients with other types of central lines (*p* = 0.034).

## Discussion

Central line type seems to play a role in CLABSI at this facility, after controlling for dwell time, a well-known risk factor for device-related infections, with implantable devices found to have a significant and sizeable positive relationship. However, literature examining the role of central line type or the area of central line placement in the development of CLABSI is extremely limited in neonates.

A 2013 study by Yumani et al. investigated central line type and CLABSI incidence, finding umbilical catheters to have a higher risk of infection then other central line types in a University Medical Center located in the Netherlands. However, this study did not specify an implantable central line type, instead identifying the following categories: UVC, CVC, and PICC.^7^ This is inconsistent with our findings, which did not identify UVCs as being an independent risk factor for CLABSI; however, in our analysis, we also included a separate category for implanted devices.

A 2010 multivariate analysis by Geffers et al. identified CVC and PICC devices to be independent risk factors for CLABSI in 22 participating neonatal departments in Germany.^4^ Similarly, Zingg et al. found PICC devices used in short duration (up to 7 days) to be an independent CLABSI risk factor in their 2010, prospective study of neonates at a facility in Switzerland.^8^ Again, these are inconsistent with our findings, where PICC and CVC devices showed no significant relationship with CLABSI. In fact, our results indicate PICC devices to be the safest in terms of CLABSI risk; however, implantable devices were not defined in these studies, either.

A 2018 study by Garcia et al. at a NICU in Mexico City, Mexico, investigated the role of central line placement area in the development of CLABSI. This study indicated, at the bivariate level, internal jugular placements were significantly more common in the group that contracted CLABSI; however, this relationship did not hold in the multivariate analysis.^1^ This is consistent with our findings; the neck site group, where internal jugular is the most common site used (59% of all neck site group placements), was found to be significantly more common in the CLABSI group in the bivariate analysis and significantly associated with increased CLABSI in the multivariate analysis. The Garcia study did not specify central line type, which means it is possible implantable devices made up a large portion of catheters being inserted into the internal jugular of patients at their facility.

In February 2022, the Healthcare Infection Control Practices Advisory Committee (HICPAC) released new guidelines for preventing CLABSI in the NICU. From their comprehensive review of the evidence related to central line type and CLABSI, they determined, “In the setting of current standard of care, the impact of prioritizing different catheter types is unknown.” As such, they recommend that central line type be dictated based on the clinical needs of the patient and not based on CLABSI prevention. However, of the cited literature, very few studies included implantable devices in their analysis and some of these did not control for dwell time.^16^ Our analysis, which controlled for dwell time, contradicts their recommendation, as implantable devices were highly implicated in CLABSI, which suggests a need to consider prevention when selecting central line type. Further research evaluating the relationship between central line type and CLABSI is warranted.

The significant link between implantable central lines and CLABSI at this facility was surprising. As a result of this finding, the Infection Prevention and Control Department is beginning conversations with involved parties regarding medical appropriateness and necessity of implantable devices, as well as possible alternatives to these devices in certain circumstances. Different areas of placement are also being explored, as conversations with NICU staff from other facilities indicated the neck site as particularly problematic for disinfection, especially if the patient is connected to other medical devices, like a respirator. Additionally, having awareness of the connection between implantable devices and CLABSI will facilitate appropriate surveillance and interventions in this specific area moving forward.

Our study has some limitations. First, while our overall sample of central line placements is sizeable *N* = 1,356), the outcome of interest within that sample, CLABSI cases, is small (*N* = 35); when CLABSI cases are matched 1:1 with similar patients, the sample becomes especially small. A small sample size can make it difficult to determine if a particular outcome is a true finding. What most commonly occurs is type II error; however, in our study, despite the small sample, our analysis was able to detect significant differences between patients contracting CLABSI and those who did not.

Additionally, our study does not specifically control for severity of illness in each patient; neonates who are more immunocompromised by the severity of their condition are more likely to contract CLABSI. Further, requiring an implantable central line may be its own marker of condition severity. We explored incorporating a neonatal disease severity score into our analysis; however, there are a variety of scoring systems, each with their own limitations, either in complexity of data required and subsequent analysis and/or limited research supporting their validity.^17^ Our analysis did control for variables that could be considered markers of condition severity, such as very low birth weight, which likely addresses some of this confounding. We are exploring other possible methods for calculating condition severity in neonates for future research.

In conclusion, in addition to commonly known risk factors for CLABSI in this NICU population, we find evidence to suggest that surgically implanted, permanent central line devices are significantly more associated with contraction of CLABSI. Research examining the relationship between central line type and area of placement and CLABSI risk in a NICU population is severely limited, and yet our findings indicate it as an area in need of further exploration.

## Research transparency and reproducibility

The deidentified dataset and Stata code used in this analysis are available upon request.

## Previous presentation

An early abstract for this project was accepted and presented at the 2023 Association for Professionals in Infection Control and Epidemiology (APIC) Conference in Orlando, Florida, in June 2023; it received the Blue Ribbon Award for exemplary scientific merit.
